# Line Managers’ Perspectives and Responses when Employees Burn Out

**DOI:** 10.1007/s10926-023-10117-3

**Published:** 2023-07-05

**Authors:** M. Claeys, A. Van den Broeck, I. Houkes, A. de Rijk

**Affiliations:** 1 Faculty of Health, Medicine and Life Science, Maastricht University, Maastricht, 6229 ER the Netherlands; 2 Department of Work and Organization Studies, KU Leuven - Campus Brussels, Leuven, Belgium; 3 Department of Social Medicine, Care and Public Health Research Institute (CAPHRI), Faculty of Health, Medicine and Life Sciences, Maastricht University, Maastricht, The Netherlands

**Keywords:** burnout, mental health, occupations, role, qualitative research, secondary prevention

## Abstract

**Purpose:**

Little is known about whether burnout can be stopped at an emerging stage. To develop this knowledge, we focus on line managers’ perspectives and responses when an employee who seems to be heading for burnout is still at work.

**Methods:**

We interviewed 17 line managers working in the educational and health care sectors, who had been confronted with the sickness absence of at least one employee due to burnout in the past. Interviews were transcribed, coded, and analyzed thematically.

**Results:**

During the period that the employee seemed to be developing burnout while still at work, line managers experienced three different, successive phases: picking up signals, role-taking, and re-evaluation. Line managers’ personal frame of reference (e.g., having experience with burnout) seemed to influence whether and how they picked up signals of burnout. Line managers not picking up signals, did not take any action. When picking up the signals, the managers however generally took an active role: they started a conversation, changed work tasks, and - at a later stage - adapted the employee’s job description, sometimes without consulting the employee. The managers felt powerless yet learned from the experience when subsequently re-evaluating the period during which employees developed symptoms of burnout. These re-evaluations resulted in an adapted personal frame of reference.

**Conclusion:**

This study shows that improving line managers’ frame of reference, e.g., by organizing meetings and/or training, may help them to detect early signals of burnout and take action. This is a first step to prevent the further development of early burnout symptoms.

**Supplementary Information:**

The online version contains supplementary material available at 10.1007/s10926-023-10117-3.

## Introduction

Burnout is classified as an occupational phenomenon by the World Health Organization [1]. It is defined as *“a syndrome conceptualized as resulting from chronic workplace stress that has not been successfully managed*” [1]. According to the Diagnostic and Statistical Manual of Mental Disorders (DSM-5), it is defined as an adjustment disorder and is characterized by overexertion and feelings of fatigue and exhaustion that have prevailed for at least six months [2]. Recently, it has been characterized by four dimensions: exhaustion (inability to perform), mental distance (unwillingness to perform), cognitive and emotional impairment; and three secondary symptoms: psychological stress symptoms, psychosomatic complaints, and depressed mood [3]. This recent characterization is an extension of the most common definition of the syndrome of burnout in terms of three dimensions: emotional exhaustion, depersonalization, and reduced personal accomplishments [4–7].

Following the job demands-resources model (JDR-model), it is assumed that when employees are confronted with excessive job demands and few resources, they are at risk of developing burnout and may be absent from work [8]. Worldwide burnout seems to be especially prevalent in the medical, health care, and educational sectors, with figures ranging around 77%, while overall burnout rates are estimated at around 8–10% [9–11]. In the county of Flanders in Belgium, where this study took place, health care providers (15.2%) and educational staff (21.1%) are also considerably more at risk than people in other sectors, where 13.6% reports burnout-related symptoms. Therefore, this study focuses on the educational and health care sectors [12].

Notably, the burnout process may unfold differently for different people [13]. For example, for men, the onset of burnout manifests as depersonalization (‘cynicism’), which evokes emotional exhaustion over time. Reduced personal accomplishment appears to develop independently. In contrast, in women, emotional exhaustion has been found to trigger burnout, which then strongly influences depersonalization and reduced personal accomplishment. Ultimately, this process likely results in sickness absence, as a way to cope with burnout symptoms [14], or other consequences such as job turnover, depression, and heart disease [3]. As line managers, also named ‘direct supervisors’, are directly involved with their employees, they may play a critical role in the development of burnout. Because of their close relationship [15], line managers should be able to recognize fluctuations in employee behavior and detect early signs of burnout [16] and subsequently prevent its further development. Belgian employers are obliged to assess the psychosocial risk (PSRs) in their company (e.g., workload, lack of autonomy) and take preventive measures to avoid burnout. However, no clear guidelines or rules are set out for line managers in relation to the prevention of burnout [17], which opens the question whether and how they are involved in preventing or stopping the emergence of burnout among their employees (e.g., by adapting the employee’s work or workplace to reduce demands and increase resources). According to the JDR-model, explaining the development of burnout, preventive actions can be include limiting the job demands or increase the job resources [8].

We aim to add to the knowledge on how burnout can be prevented by examining the perspectives and responses of line managers regarding employees developing early signs of burnout, a topic which has not received much research attention thus far. Specifically, we aim to answer the following research questions: (1) What are line managers’ perspectives on the early development of burnout in their employees; and (2) How do line managers’ respond to early signs of their employees developing burnout to reduce the risk of them dropping out. To be sure line managers’ expressed insight about burnout in their employees, the employees should have been in sickness absence and returned to work due to burnout. We focus on their perspectives and actions to reduce the risk of their employees becoming burned out.

## Method

### Methodological Orientation and Theory

We took a thematic qualitative analysis approach [18], which takes theory as a starting point. The theories used were that for burnout [1–3] and the JDR-model [8]. The latter assumes that employees are at risk of developing burnout when they experience too many job demands and too few job resources [8].

### Design

We used a qualitative research design to understand the line managers’ perspectives and actions better regarding employees developing burnout.

### Setting and Participants

The recruitment took place between March and May 2021. To include line managers with a wide range of views and perspectives, we used convenience and purposive sampling. Line managers could be included if they (a) worked in the educational or health care sector, (b) had been directly involved in the return to work (RTW) process of an employee who had suffered from burnout between two and five years ago, to capture relatively recent experiences but exclude cases that occurred during the COVID-19 pandemic, as the amount of telework may have impeded their noticing early burnout symptoms, and (c) were willing to discuss real-life cases regarding the process prior to sickness absence, due to burnout, of their employee. Before the interview, participants were only informed about the inclusion criteria and asked whether they would be willing to talk about their experiences and responses towards the employee who developed burnout, yet they did not receive the interview guide or the specific themes that were included therein. One participant was sampled via a flyer on ‘Facebook’, while 17 managers were found by sharing the information in the authors’ networks. Two of the latter participants did not meet the inclusion criteria and were removed. In addition, 53 schools and health care organizations were approached by e-mail. This resulted in one participant and two exclusions. Non-responses were due to a poor fit with the inclusion criteria or lack of time or motivation to participate in the research. The final sample included 17 line managers.

**Table 1:** Participants’ characteristics

	Age	Gender	Sector	Experience line manager	Experience burnout in employee	Headmaster/director
**1.**	55	F	Health care	Senior	Multiple	
**2.**	43	M	Health care	Intermediate	Multiple	
**3.**	48	F	Education	Junior	Multiple	X
**4.**	44	F	Education	Senior	Multiple	X
**5.**	55	F	Education	Senior	1	
**6.**	45	F	Education	Intermediate	Multiple	X
**7.**	62	F	Health care	Senior	Multiple	
**8.**	56	F	Health care	Senior	Multiple	X
**9.**	46	F	Education	Senior	Multiple	X
**10.**	45	F	Health care	Senior	Multiple	
**11.**	60	F	Education	Senior	Multiple	X
**12.**	NK*	F	Education	Intermediate	2	X
**13.**	45	M	Health care	NK*	1	
**14.**	42	F	Health care	Junior	1	
**15.**	56	F	Education	Senior	1	X
**16.**	43	M	Health care	Senior	2	
**17.**	46	F	Health care	Senior	1	

Gender; F: female/ M:male Experiences line manager; Junior: 0–5y/Intermediate:5–10y/Senior:+10y *Not known

The final sample consisted of 14 female and three male participants (see Table 1). Participants had an average age of 49 years. Eight of them shared their experiences working in the educational sector. The other nine participants shared experiences in the health care sector. Nine out of the 17 were employed in an additional function (director, headmaster), but all spoke from their experience as being directly involved in the burnout process as a line manager. The total length of experience of the participants as a line manager varied from 1 to 5 years up to more than 10 years. All participants had experience with at least one employee becoming burned out.

### Data Collection

The semi-structured in-depth interviews (mean duration of 51 min) took place via the online communication platform the participants preferred (e.g., Zoom) by one of the two female interviewers (MC, LV), doing a Masters’ degree in Occupational Health and Business Administration or Work, Health, & Career. The interview guide (see Table 2 for topics) was developed by MC, LV AVdB, and AdR, and based on the literature and the research question, and was not pilot tested. Since this study is part of a larger study, the interview guide exceeds our research questions. However, some information given in response to interviews items related to other phases in the burnout process, helped the researchers to better understand the case and interpret the information related to the research question in this study.

**Table 2:** Semi-structured interview guide

*Part of the interview*	*Items and questions*
*Start*	Explaining the goal of the research, anonymity, confidential treatment of the interview data
*Background information*	• Inclusion criteria • Age and gender • Function • Information about burned out employee(s) • Type of leadership style
*Questions of period of burnout development*	• Perceived burnout signals • Perceived causes of burnout of their employees • What happened between perceiving the signals and sickness absence period
*Questions on sickness absence period*	• Communication/contact during that period • Persons involved • What happened with the tasks of that employee during that period • Experiences during sickness absence period of employees
*Questions on RTW period*	• First conversation about RTW • RTW process • Who was involved during the RTW • Arrangements • Important aspect of leadership style
*Ending the interview*	• Intentions to change approach in the future • Learning effect • Additional concerns about the discussed topic
Thank you!	

### Data Analysis

After transcribing and pseudo-anonymizing the interviews, a thematic qualitative analysis took place [18], using the Qualitative Analysis Guide of Leuven (QUAGOL) [19], which includes the following steps: bracketing, (re)reading the transcripts, and summarizing the manuscripts in a narrative report (+/- 2 pages/interview). Transcripts were not returned or checked to reduce the burden for the participants. N-Vivo was then used to conduct the actual coding process, which included open coding, formulating themes (derived from data) and the analysis of the relationships between themes using constant comparison and analytical induction. This eventually led to a conceptual scheme, the code-tree. The code-tree was produced by N-Vivo. As the code-tree overlaps with how the results were presented, the code-tree is not presented here. During the coding process, transcripts and summaries were (re)read as many times as necessary to refine the concept list, and each step was discussed with a senior researcher. Two data coders were involved in this process (MC and AdR). Data saturation was reached at the level of experiences. This article followed the criteria of the Consolidated criteria for Reporting Qualitative research (COREQ) [20].

## Results

**Fig. 1:**
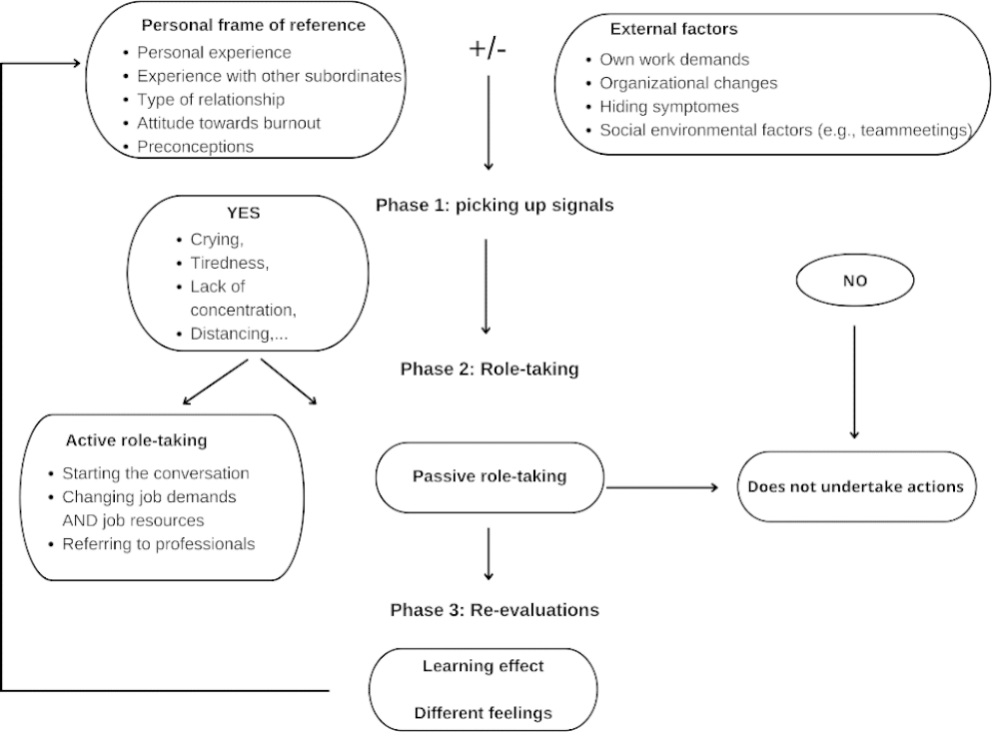
Line managers’ experiences and role-taking

The line managers varied in the degree to which they consciously took a role in managing the process of preventing burnout among their employees. First of all, they may or may not have picked up the burnout signals of their subordinates, depending on their personal frame of reference. Those who did not observe any signals were self-evidently not able to take any action in response. If the managers noticed the signals, they took a rather passive or a more active role in order to prevent further development of the burnout. During the re-evaluation of the past experience, the line managers expressed feelings of powerlessness (see Fig. 1).

### Phase 1: Picking up Signals

Whether managers took action in relation to burnout was first of all determined by whether they picked up the signals or not.

Only a few line managers stated not having observed any signals related to the burnout development, not even after the employee reported sick.

*“But it is only because of other people saying, ‘Oh, it doesn’t run well there’, that you start to pay attention and then, especially afterwards, you start to look back and then I think, oh yes, that was actually a signal and I didn’t actually see it.” (participant 3)*

According to the line managers, this was primarily because burnouts mostly took place among perfectionists, who were highly engaged in their work and had stable personalities. The line managers did not expect such engaged people to develop burnout.

*“Very positive, a very enthusiastic person. Typical also that you don’t see it coming.” (participant 14)*

Additionally, the line managers suggested that being befriended with the employee masked the burnout signals.

*“Yeah, that one was a little stronger, because she’s part of a little group, um, where we meet outside of work every year anyway. And that is, I only have 1 such group and she is in it. .... From the moment she realized she was on sick leave, I didn’t have the idea that it was burnout. At that time it was not clear to me.” (participant 15)*

Line managers also expressed difficulty in picking up the signals because they believed that employees often tried to hide their burnout symptoms or did not recognize their own signals.

*“It is only in retrospect that all this became clear. For her as well, because she had not felt it (the burnout) coming.” (participant 17)*

Additionally, other line managers blamed their own job demands or organizational changes in the period in which the burnout signals manifested.

*“Because if it’s very busy, I’m actually almost never there (teachers’ room). So then I also see less of how it is with the people.” (participant 15)*

*“And I had made her responsible for the group and that gave her a boost, but when I look back at it now, I think that it must have started somewhere (since she changed job).” (participant 2)*

While this indicates that some managers did not recognize any burnout symptoms due to various reasons, the majority of line managers in our sample did notice some early burnout signals among their employees. Often they noticed that the employees could not control their emotions anymore, had difficulty concentrating, or lost their self-confidence and started to question their role as employee, partner, and parent.

*“The way she drafted her emails, even to colleagues. She was very gruff, not what she was before. ” (participant 10)*

*“For instance, when we were having a meeting, often, there were tears.” (participant 16)*

Managers suggested that employees felt limited in their functioning, expressed the need to go on vacation, and showed tiredness. Employees had difficulty dealing with extra tasks and were more frequently absent from work.

*“Being fed up, huh, being tired, not sleeping, bags under her eyes, yes. You can see that something more than just not sleeping well for a while is going on there.” (participant 5)*

On top of that, some managers also noticed the employees distanced themselves from their colleagues and from the line mangers.

*“She distanced herself very strongly from me.” (participant 2)*

Several factors acted as a facilitator to picking up these behaviors as signals of developing a burnout.

One’s personal frame of reference and, more specifically, one’s own experience with burnout (e.g., their own or previous burnout cases at work or in their private life) and recognizing burnout as a serious mental health disorder (e.g., accepting attitude) acted as a facilitating factor to pick up the burnout signals. Their earlier experiences increased the line managers’ awareness and knowledge about the burnout signals. Additionally, those line managers tended to have a more accepting attitude towards burnout. According to the interviewees, this helped to create a more open culture at work in order to disclose any issues related to burnout. Therefore, employees were less prone to hide their burnout symptoms and were facilitated to be open about it.

*“Usually, they do speak openly to me. I don’t know if that’s because they know I’ve had a burnout and that makes them more open, that’s also possible. So I do find out very quickly, WOW this is not going well with them....I am very understanding of that but of course, that is because I have had it myself.” (participant 1)*.

In addition, social environmental factors (e.g., help from other subordinates and team meetings) were also related to recognizing burnout signals.

*“Yes, because we have a weekly team meeting, we actually saw it coming as a team.” (participant 12)*

### Phase 2: Role-taking

Line managers needed to pick up the signals in order to engage in role-taking to prevent the further development of burnout.

Some line managers expressed that they adopted a passive role. Despite being aware that their employee was tending towards burnout, they did not intervene as they did not feel responsible and/or capable in their role as line managers to do so or were unaware that they could have meaningfully intervened to further prevent burnout.

*“No, because I think I’m not a therapist and I can’t get into that mixed role either. People can vent, and I find that very healthy but I really guard against giving advice myself.” (participant 6)*

However, the majority of the interviewees noticing the burnout signals, engaged in an active role. This was mainly related to their accepting attitude to burnout (e.g., recognizing burnout as a serious mental health disorder): they had experienced burnout themselves and/or wanted the best for their employees and felt responsible for that. Additionally, some of these line managers had been trained in burnout prevention and tried to put into practice what they had learned.

In terms of role-taking, these line managers indicated that they first started a conversation with the employee developing burnout. During this conversation, they aimed to increase the employee’s insights into his or her burnout development.

*“Yes, we do have a several people every year that we feel are heading towards burnout and whom you try to bring to understanding.” (participant 8)*

They tried to reassure the employee, show that they were there for them and offered help. They were keen to receive information about what was going on. This is much in line with their assertion that they wanted to be a democratic coach and people manager.

*“Making people feel like it’s okay, okay who you are. You can try to change things, but you are who you are.” (participant 12)*

Some line managers assumed that having a good relationship facilitated starting the conversation. Yet, none of the participants could report an experience with burnout in an employee they did not have a connection with.

*“I don’t know whether it would be different if I were to have more or less distance from her. I don’t know that. It’s fascinating though.” (participant 14)*

Taking an active role in preventing the further development of burnout included changing the work in consultation with those employees expressing burnout signals. Mostly, the line managers tried first to obtain sufficient information before making changes to the job, yet often already started to advise how the work tasks could be changed during the first conversation.

“*And then you notice that when you talk to people, they sometimes indicate things, and if we can, we try to make adjustments in their tasks. That has already happened, yes. As a result, there has been no burnout.” (participant 11).*

When this first set of actions did not work, line managers felt the need to take more drastic actions.

*“At a certain point we also said to our colleague that we should stop here for a managerial position. So much so that you say, ‘OK, this is definitely not the solution for you, you have to get out of here because you’re going to make yourself unhappy, and you’re going to have to give up.” (participant 8)*

First, they engaged in a more directive role by changing work tasks without discussing this with the employee.

*“So that’s always a struggle though, and at some point, you do have to cut things and start taking away certain things.“ (participant 4)*

Second, they advised the employee with burnout signals to visit a psychologist or their general practitioner. They thus openly and directly indicate that there is a problem. However, many line managers hesitated to take such actions.

*“Then at some point I also advised that it was best that she makes an appointment with the general practitioner.” (participant 16)*

Line managers – particularly those taking an active role – indicated experiencing different feelings about their role-taking. Primarily, powerlessness was a commonly discussed feeling among the line managers.

*“So, that makes you feel agitated, because you think the job is what it is. I cannot change something on that level.” (participant 14)*

Additionally, they feel ignorant and insecure about their role-taking: questioning if they could not have done more or done something differently.

Finally, some line managers decided to engage in an active role even before burnout signals occurred. As there were no signals yet, the measures taken were generic and could be applied to any employee within the organization or department.

*“The team learn to take cues from each other. I say if you notice that someone is disturbing you, please come and say so. Do not just walk around with that, come and say so. If someone starts behaving abnormally, or suddenly gets very emotional, or... We should be able to discuss it. That’s not always obvious.” (participant 17)*

### Phase 3: Re-evaluation

Ultimately, all of the discussed cases ended up taking sickness leave due to burnout. For the line managers, the time came to process the past period and to re-evaluate if they could have done more to learn lessons to prevent sickness absence due to burnout in the future.

First, they indicated having felt powerless, as they were not able to invest as much time as they wanted and/or because they were unable to recognize the signals. These reflections also contributed to not feeling responsible regarding the development of burnout.

*“Those are things you can’t change, huh? And that’s the hardest thing. If adjustments can be made, then I’m open to that, but it’s not always possible. Hey, and then you are backed into a corner. ” (participant 15)*

Other line managers felt guilty, particularly when the link with the working conditions was made. They questioned themselves and their role-taking.

*“The human aspect is very important to me. People need to feel good in order to function well. Being so close to someone without noticing something. It made me think, oops, what is going on here? What did I miss? How did I not notice it? Yes, you do question yourself about it.” (participant 17)*

Despite that not all of them felt responsible, line managers tried to learn from their experiences. They confirmed that they had gained more understanding and knowledge of, for example, the burnout signals and developments. They expressed the intention to handle the situation differently next time.

*“I always try to keep in mind what we are already doing as an organization. But I do try to look out for what else can we do?” (participant 11)*

*“Yes, not having a judgement ready too fast. That is important. Also, recognizing the signals is very important and making issues discussable, talking, communicating. Sometimes though, you don’t wish to talk about it, hey, because you see someone and you’re thinking, oh no, another one who’s going to take absence. But not talking about it is sometimes worse because the sooner you can intervene, the more chance you’ve have that someone doesn’t have to take absence.” (participant 1)*

Those who did not perceive signals remained astonished.

*“Usually, I’m amazed that I didn’t see it coming.” (participant 11)*.

However, during the sickness absence and RTW period of the employee, those line managers who were unaware of the burnout developing gained more information about what happened and how they could prevent the development of burnout in future cases. This made it a learning experience for those line managers as well.

In summary, managers who had dealt with burnout themselves and who had an accepting attitude towards burnout were more likely to pick up the signals of early burnout (e.g., getting emotional, not able to handle any more tasks). They engaged in different roles depending on the recognition of the burnout signals. Their role-taking was either passive (not doing anything) or active (e.g., starting the conversation, changing job descriptions) to prevent the burnout from developing further. While re-evaluating their experiences, all line managers formulated the intention to deal better with future situations (e.g., pay more attention to the signals and try to react faster to them) than they had done previously.

## Discussion

This study aimed to gain a better understanding of the perspectives and actions of line managers during a period in which one of their employees developed burnout, with theory on burnout [1–3] and the JDR-model [8] as basis for the data collection and analysis. The results revealed three phases in relation to reacting to symptoms of burnout and dealing with the employees’ demands and resources. First, recognizing signals is the essential starting point of line managers’ actions and the most important prerequisite for the managers’ engagement in an active role. It is facilitated by the managers’ personal frame of reference. The main signals perceived by the line managers are remarkably in line with the four burnout dimensions put forward most recently by Hadžibajramović, Schaufeli, and De Witte [3]. The most frequently observed burnout symptoms in this study were exhaustion and emotional impairment [13]. Additionally, managers recognized cognitive impairment and mental distance [3], which likely lead to reduced personal accomplishment, as mentioned in the older definition of burnout [4]. It is not clear whether the line managers were influenced by the literature and training for burnout or whether the current results could be regarded as a confirmation of the validity of the existing definitions of burnout [3, 4].

The second phase was role-taking. We found both passive and active role-taking managers in addressing early symptoms of burnout. Our results thus indicated that receiving help when developing burnout cannot be taken for granted. The literature indicates that stigma, shame and fear about needing care may prevent a clear communication between line managers and employees [21, 22]. Whether or not employees seek help thus depends on the professional accessibility of the potential help provider (i.e., line managers) and their trust in them. However, since line managers often have multiple subordinates and many job demands, we could question whether recognizing burnout is feasible for them, especially when their job involves a wide scope of control. Having a good relationship with one’s employees is helpful in giving support and starting to take action [23]. Notably, some line managers did not notice any burnout signals from their employees. This may be due to their own job demands because a caregiver should have sufficient resources to provide that care [24].

Most line managers acknowledged burnout as a serious mental health disorder. This is in line with studies investigating managers’ negative attitudes towards employees with depression [25, 26], which concluded that more highly educated female managers working in the public sector are less likely to report negative attitudes regarding burnout compared to less educated male managers working in the private sector.

When line managers noticed the burnout symptoms, most of them took action to prevent the further development of the burnout. The results suggest two sets of actions. The first set of action focused mainly on balancing the job demands and resources with the consensus of the employee [27]. While changing the employee’s tasks was mostly done as an attempt to decrease the job demands, talking with the employee about their worries for his/her health can be seen as an increase in the job resource of social support, which is an important buffer for burnout [28].

Though most line managers described their leadership style as democratic, people manager, and coaching, some line managers changed to a more paternalistic or even directive leadership style by taking action (second set of actions). This may suggest that line managers engaged in a situational leadership style or that they – in the case of stressful situations – may be unable to sustain their more people-oriented style.

Some line managers, who did pick up the signals, tried to avoid their responsibilities and engaged in passive role-taking. They attributed the burnout to their employee’s personalities or private life. However, burnout is work-related, at least partially [1, 3, 4, 27], which implies that line managers do have responsibilities. According to Belgian legislation, employers are obliged to undertake preventive action to prevent psychosocial risks at work, and they are also expected to engage in harm reduction [29]. However, there are no guidelines or obligations set for them.

The third phase focused on re-evaluation. Regardless of acting actively or not, the line managers felt powerless regarding balancing their interest in the organization and their duty towards the employees [30]. Similar struggles have been reported in research examining employers who are confronted with employees with a cancer diagnosis [30]. Also, the line managers who perceived the burnout as not work-related still felt powerless. Yet they did not feel not responsible for ‘solving’ or preventing its further development.

Finally, the line managers said they learned from their experiences, which added to their personal frame of reference. It encouraged them to set the intention to act in a better way (e.g., pay more attention to signals, try to engage in an active role faster) when facing another employee developing burnout. According to the literature, learning through experience enhances people’s skills, self-efficacy and might help overcome barriers. However, guided practices are preferred [31].

### Methodological Strengths and Limitations

This study has several theoretical and methodological strengths. It adds to the paucity of research on the role of line managers in the burnout development process and used the QUAGOL guide, in which one engages in constant data comparison during data analysis. This increases the trustworthiness and transparency of this research. Bracketing eliminated personal interests while analyzing the data [19]. Data saturation was reached at the level of experiences.

However, this study is also subject to some limitations, including selection bias. First, all participants were informed their employees went on sickness absence due to burnout. Second, all interviewees were recruited in the network of the researchers and participated voluntarily. They might have been more willing to support their employees than the average line managers and be more open about it. Our study might thus not fully cover the perspectives and actions of line managers of whom the employees did not reveal the reasons for their sickness absence of managers who neglected the burnout development in their employees. Third, this study only contains ‘failed preventive cases’. Therefore, we could not study the efficacy of potential preventive actions for the further development of burnout. Hence, it does not provide information about employees with early symptoms who did not develop a burnout. Fourth, the majority of participants were female. Female managers are overrepresented in the health care and education sector, but have been found to take mental health disorders more seriously [25, 26]. Yet, the sample represented sufficient variety to answer the research question: the sample represented 17 different organizations in health care and education and the participants varied in working experience.

### Recommendations for Further Research

Future studies should focus on the needs of the line managers in order to prevent burnout development in their employees. Therefore, it could be interesting to study the effects of training line managers in recognizing of and reacting on the development of burnout symptoms among their employees. Research on preventive actions [32] is needed to know what is effective in preventing burnout. While decreasing job demands and increasing job resources is suggested to be effective, little is known about the effects of holding conversations to confront employees with their burnout symptoms. Our study suggests that the managers’ frames of reference influenced their experiences and responses when their subordinates develop burnout. Future research could study potential personal factors affecting the experiences and responses of managers, including their gender and seniority, as well as contextual elements (e.g., their own demands and resources or organizational culture) that may have an impact. In addition, research in other occupational sectors is warranted, as male-dominated sectors might bring about different attitudes and perspectives on burnout. Additionally, as hybrid work may challenge the early detection of burnout, future research may focus on the line managers’ perspectives and actions within such a context. At last, future research could investigate whether situational leadership, which may unable to sustain a more people-oriented style, is beneficial when employees start to develop burnout.

### Practical Recommendations for line Managers and Organizations

Based on our results, it can be recommended to train managers further in taking preventive actions, recognizing the signals, and taking adequate steps to avoid burnout. Such training courses should focus on real experiences and intervision [31, 33] to contribute to the managers’ personal frame of reference. These intervisions could also improve the line managers’ sense of responsibility and facilitate them to engage in an active role in preventing burnout even though they may attribute the burnout to factors outside the occupational context. In addition, employees often struggle to ask for help since they fear mental illness-related prejudice and discrimination from their line managers and their colleagues [34, 35]. Hence, reducing the organizational stigma on struggling with high demands could help both employers and line managers to prevent burnout. Finally, our results may provide preliminary guidelines on how to take action, which could support line managers during the burnout development period in their employee.

## Conclusion

Managers need to pick up the burnout signals before they can actively help employees with burnout symptoms. Different factors in the line managers’ personal frame of reference (i.e., personal experiences, an accepting attitude, and the kind of relationship with their employee) influenced picking up signals to a certain extent. Social environmental factors influenced this recognition as well. When engaging in a passive role, no actions are taken. Active role-taking consists of different phases to decrease job demands and increase job resources. The re-evaluation conducted by the line managers enlarged their personal frame of reference, leading to their intention to engage in active role-taking in future burnout cases.

### Electronic Supplementary Material

Below is the link to the electronic supplementary material.

Supplementary Material 1

## Data Availability

The datasets generated during and/or analysed during the current study are available from the corresponding author upon request.
