# Supplementary material for: Line Managers’ Perspectives and Responses when Employees Burn Out
Source: J Occup Rehabil. 2023 Jul 5;34(1):169–79. doi: 10.1007/s10926-023-10117-3 (PMC10899266; doi:10.1007/s10926-023-10117-3)
Supplement: Supplementary file 1 — Supplementary Material 1 [file 10926_2023_10117_MOESM1_ESM.docx]

Line managers’ perspectives and responses when employees burn out

**Authors:** M. Claeys*, A. Van den Broeck**, I. Houkes***, A. de Rijk****

*Manon Claeys- Faculty of Health, Medicine and Life Science, Maastricht University, 6229 ER Maastricht, the Netherlands- [manon.claeys@hotmail.com](mailto:manon.claeys@hotmail.com)

** Anja Van den Broeck - Department of Work and Organization Studies, KU Leuven - Campus Brussels, Belgium. – [Anja.vandenbroeck@kuleuven.be](mailto:Anja.vandenbroeck@kuleuven.be)

***Inge Houkes- Department of Social Medicine, Care and Public Health Research Institute (CAPHRI), Faculty of Health, Medicine and Life Sciences, Maastricht University, Maastricht, The Netherlands- [Inge.houkes@maastrichtuniversity.nl](mailto:Inge.houkes@maastrichtuniversity.nl)

****Angelique de Rijk - Department of Social Medicine, Care and Public Health Research Institute (CAPHRI), Faculty of Health Medicine and Life Science, Maastricht University, The Netherlands- [Angelique.derijk@maastrichtuniversity.nl](mailto:Angelique.derijk@maastrichtuniversity.nl)
